# The Electrochemical Detection of Bisphenol A and Catechol in Red Wine

**DOI:** 10.3390/foods14010133

**Published:** 2025-01-06

**Authors:** Chao Wang, Xiangchuan Wu, Xinhe Lin, Xueting Zhu, Wei Ma, Jian Chen

**Affiliations:** 1 School of Biotechnology, Jiangnan University, Wuxi 214000, China; wangchao@jiangnan.edu.cn (C.W.); wei.ma@jiangnan.edu.cn (W.M.); 2 Science Center for Future Foods, Jiangnan University, Wuxi 210023, China; 3 School of Food Science and Pharmaceutical Engineering, Nanjing Normal University, Nanjing 210023, China; wxyaxxx@163.com (X.W.); 18068849373@163.com (X.L.); 13913931306@163.com (X.Z.)

**Keywords:** nanozyme, laccase, electrochemical detection, bisphenol A, catechol

## Abstract

The use of nanozymes for electrochemical detection in the food industry is an intriguing area of research. In this study, we synthesized a laccase mimicking the MnO_2_@CeO_2_ nanozyme using a simple hydrothermal method, which was characterized by modern analytical methods, such as transmission electron microscope (TEM), X-ray diffraction (XRD), and energy dispersive X-ray spectroscopy (EDX), etc. We found that the addition of MnO_2_ significantly increased the laccase-like activity by 300% compared to CeO_2_ nanorods. Due to the excellent laccase-like activity of the MnO_2_@CeO_2_ nanozyme, we developed an electrochemical sensor for the detection of hazardous phenolic compounds such as bisphenol A and catechol in red wines by cyclic voltammetry (CV) and differential pulse voltammetry (DPV). We used the MnO_2_@CeO_2_ nanozyme to develop an electrochemical sensor for detecting harmful phenolic compounds like bisphenol A and catechol in red wine due to its excellent laccase-like activity. The MnO_2_@CeO_2_ nanorods could be dispersion-modified glassy carbon electrodes (GCEs) by polyethyleneimine (PEI) to achieve a rapid detection of bisphenol A and catechol, with limits of detection as low as 1.2 × 10^−8^ M and 7.3 × 10^−8^ M, respectively. This approach provides a new way to accurately determine phenolic compounds with high sensitivity, low cost, and stability.

## 1. Introduction

Phenolic compounds in wine are known to have antioxidant properties, preventing certain diseases [1,2]. However, some phenolic substances can be harmful and affect the safety and taste of wine [3,4]. For example, bisphenol A (BPA), an endocrine disruptor, may migrate to wine from plastic packaging [5,6,7]. Additionally, catechol (CC) can contribute to bitter taste in wine [8]. Therefore, there is a need for a cost-effective and simple method for identifying and quantifying these harmful phenolic compounds. Traditional testing methods such as gas chromatography [9], liquid chromatography [10], and capillary electrophoresis [11] are expensive and require complex operations, limiting their application for in situ detection.

Laccase, which can catalyze the oxidation of a wide range of phenolic compounds [12,13,14], has been considered a candidate for detecting phenolic compounds [15,16]. However, its application has been hindered by low stability, high cost, and poor reusability [17,18,19,20,21]. In recent years, nanozymes, which are inorganic nanomaterials with intrinsic enzyme-like catalytic activity, have received increasing attention due to their high stability, low cost, cyclic use, and good polyfunctionality [22,23,24,25]. Consequently, various nanomaterials have been discovered, such as carbon nanomaterials [26,27], metal (hydrogen) oxides [28,29], metal chalcogenides [30,31], and precious metal nanomaterials [32,33].

As alternatives to natural enzymes, nanozymes have been extensively utilized in biosensing, bioimaging, antimicrobial applications, antioxidant therapeutics, and environmental remediation [34]. A variety of nanozymes have been developed using metal-based elements (such as Ti, V, Cr, Mn, Fe, Co, Ni, Cu, Zn, Zr, Mo, Ru, Rh, Pd, Ag, Cd, Sn, Sb, Ce, Hf, Pt, Au, Bi), non-metal-based elements (such as C, B, Se), and their compounds or heterocomplexes [35,36]. The chemical composition (e.g., metal-based versus non-metal-based), synthesis method (e.g., impregnation, co-precipitation, deposition–precipitation, hydrothermal/solvothermal), and physical morphology (e.g., spherical, rod, cyclic, and hollow structures) each influence the properties of nanozymes in distinct ways [34]. Cerium-based nanomaterials have garnered significant attention due to their remarkable enzyme-like catalytic activity. These cerium-based nanozymes have found applications in immunoassays, analytical detection, and free radical protection owing to their high stability, low cost, ease of synthesis and modification, and biocompatibility [37,38]. Cerium oxide, a notable nanozyme, has been increasingly integrated with various nanomaterials as research advances. For instance, Bhagat et al. reported a gold-cerium dioxide core-shell structure exhibiting excellent peroxidase, catalase, and superoxide dismutase activities [39]. Additionally, Zhu et al. developed boron nitride quantum dot-anchored porous CeO_2_ nanorods (BNQDs/CeO_2_) that effectively catalyze the oxidation of 3,3′,5,5′-tetramethylbenzidine (TMB) by H_2_O_2_, facilitating the detection of kanamycin in environmental and food samples [40].

Nanoceria, one of the important rare earth oxides, have recently attracted considerable interest due to their excellent redox ability, unique optical properties, and chemical stability [41,42,43,44,45,46,47]. Nanoceria have been reported to exhibit enzyme-like activity due to the reversible Ce^3+^/Ce^4+^ redox pair and oxygen vacancies on the surface [48]. Kailashiya et al. [49] explored the effects of nanoceria on human platelet functions and blood coagulation, while Singh et al. reported a remarkably active CeVO_4_ nanozyme that functionally mimics cytochrome c oxidase, the terminal enzyme in the respiratory electron transport chain, by catalyzing a four-electron reduction of dioxygen to water [50]. However, most nanoceria nanozymes have focused on mimicking peroxidase [14], superoxide dismutase [51], and oxidase [52], and to date, there has been scarce study on comparing the response between this nanozyme and laccase. Several laccase-like nanozymes have been reported, including Cu/GMP as described by Hao et al. [53]. However, this system requires the removal of the solid catalyst through centrifugation post-reaction, necessitating a complex process and specialized equipment. Additionally, the CA-Cu nanozyme reported by Xu et al. exhibits poor stability under extreme pH and temperature conditions, with its catalytic performance declining significantly at pH levels below 5 or above 8 [54].

In this study, we utilized MnO_2_-doped CeO_2_ nanorods (CeO_2_ NRs) to create hybrid nanocomposites with laccase-like activity, which were employed for detecting phenolic compounds. CeO_2_ NRs with well-defined reactive planes [55,56] were easily synthesized via a solution-based hydrothermal method [57]. It has been suggested that nanorods possess higher oxidation activity than CeO_2_ nanoparticles due to their more reactive planes [58]. MnO_2_ was selected as a suitable dopant to modify CeO_2_ NRs, resulting in MnO_2_@CeO_2_ NRs. The introduction of MnO_2_ induced more oxygen vacancies [59], and their strong redox behavior (Ce^3+^/Ce^4+^ and Mn^2+^, Mn^3+^, and Mn^4+^) as well as the synergistic interaction between them could also accelerate oxidation reactions [60].

This study introduces an innovative approach by developing a MnO_2_@CeO_2_ nanozyme that mimics laccase activity, enhancing the electrochemical detection of phenolic compounds. The integration of MnO_2_ into CeO_2_ nanorods significantly boosts their catalytic performance, increasing laccase-like activity by 300%. Leveraging this high activity, a novel electrochemical sensor was created for the rapid and sensitive detection of hazardous phenolic compounds like bisphenol A and catechol in red wine. By modifying a glassy carbon electrode with polyethyleneimine, the sensor achieves ultra-low detection limits, offering a highly sensitive, cost-effective, and stable method for food safety analysis. This method represents a significant advancement in the use of nanozymes for foodborne contaminant detection.

## 2. Materials and Methods

### 2.1. Reagents, Characterization Techniques

Ethanol, Guaiacol, 2,4-Dichlorophenol (2,4-DP) and 4-Aminoantipyrene (4-APP) were bought from Shanghai Sinopharm Chemical Reagent Co., Ltd., Shanghai, China. BPA, CC, KMnO_4_, CeCl_3_·7H_2_O, and polyethyleneimine (PEI) were purchased from Shanghai Aladdin Biochemical Technology Co., Ltd., Shanghai, China. Exhibiting a K_m_ value of 0.4 mM and a V_max_ value of 3 µM, laccase was obtained from Shanghai Yuanye Bio-Technology Co., Ltd., Shanghai, China. In this study, all chemicals were analytically pure, and the laccase used was a purified enzyme solution derived from Trametes versicolor. The methanol and formic acid used in the chromatographic analysis were of HPLC grade, purchased from Shanghai Macklin Biochemical Co., Ltd., Shanghai, China.

Electrochemical curves were measured by an electrochemical workstation (Chen Hua Instruments Co., Shanghai, China), using different signal transducers of cyclic voltammetry (CV) and differential pulse voltammetry (DPV). Transmission electron microscopy (TEM) images were measured by a TEM (Hitachi High-Technologies Co., Ltd., Tokyo, Japan). X-ray diffraction (XRD, Shimadzu Enterprise Management China Co., Ltd., Tokyo, Japan) patterns were recorded on the X-ray powder diffractometer. An energy dispersive spectrometer (EDS, Shanghai Jingke Scientific Instrument Co., Ltd., Shanghai, China) was used to characterize the MnO_2_@CeO_2_ nanozyme together with TEM. Absorption spectra were performed on a UV-vis spectrophotometer (UV-1800, AoYi Instruments Shanghai Co., Ltd., Shanghai, China). An enzyme-labeled instrument was provided by Gene Co., Ltd., Hongkong, China.

### 2.2. Synthesis of MnO_2_@CeO_2_ Nanozyme

The MnO_2_@CeO_2_ nanozyme was synthesized according to a previous report with some modifications [61]. Mn^4+^-doped CeO_2_ NRs with various content of Mn^4+^ (4, 8, 12, and 16 at. %) were prepared by the hydrothermal synthesis method.

In a typical synthesis, for 8% Mn^4+^-doped CeO_2_ NRs, 0.4 g CeCl_3_·7H_2_O and 0.012 g KMnO_4_ was dissolved in 30 mL of 9 mol/L NaOH solution under vigorous stirring. The suspension was transferred to a 50 mL Teflon-lined stainless-steel autoclave and held at 140 °C for 48 h. After the autoclave was cooled to room temperature naturally, fresh precipitates were separated by centrifugation and washed with deionized water to neutrality and with ethanol several times. The MnO_2_@CeO_2_ nanozymes were obtained by drying the precipitates at 60 °C overnight.

### 2.3. Evaluation of the Catalytic Performance of Nanozyme and Laccase

The catalytic activity of the MnO_2_@CeO_2_ nanozyme and laccase was determined using the colorimetric reaction between 2,4-DP and 4-APP; 2,4-DP (0.1 M, 10 µL) and 4-APP (0.1 M, 10 µL) was mixed with MnO_2_@CeO_2_ nanozyme aqueous dispersion (1 mg/mL, 80 µL) or laccase (10 mg/mL, 60 µL). Then, Tris-HCl buffer (0.1 M, pH 7.0, 180 µL) was added in the mixture up to 200 µL. The reaction was maintained at 37 °C for 2 h, and then, the absorbance was detected at 485 nm.

### 2.4. Determination of the Catalytic Kinetic Parameters

The prepared MnO_2_@CeO_2_ nanozyme (1 mg/mL, 80 μL) or laccase (10 mg/mL, 60 μL) was mixed with 2,4-DP (0.1 M, 0.05, 0.1, 0.2, 0.4, 0.6, 0.8, 1.0, 2.0, 4.0, 6.0, 8.0, and 10.0 µL) and 4-APP (0.1 M, 15 µL). Tris-HCl buffer (0.1 M, pH 7.0, 180 µL) was added in the mixture up to 200 µL. At 37 °C, the kinetics of the reaction can be determined by monitoring the change in the absorption wavelength at 485 nm over time by ultraviolet-visible spectroscopy. All the experiments were repeated thrice.

The kinetic parameter was calculated via Equation (1):

(1) $$v=\frac{V_{max\left[S\right]}}{K_{m+\left[S\right]}}$$

*v* is the initial velocity of the reaction, *V_max_* stands for the maximal reaction velocity, *K_m_* is the Michaelis–Menten constant, and [*S*] is the concentration of substrate. When calculating *K_m_* and *V_max_*, the conversion form of the Michaelis–Menten equation can be used, which is named the Lineweaver–Burk Equation (2):

(2) $$\frac{1}{v}=\frac{K_{m}}{V_{max\left[S\right]}}+\frac{1}{V_{max}}$$

### 2.5. Evaluation of the Stability of Catalyst

The MnO_2_@CeO_2_ nanozyme or laccase was incubated at varying pH (3.0–9.0) for 7 h to evaluate the effect of pH on catalytic activity. In order to study the temperature stability of the MnO_2_@CeO_2_ nanozyme and laccase, they were stored at −18~120 °C for 45 min before determining their catalytic activity. The catalytic activity at 30 °C was used as a reference. In the same way, the effect of organic solvents on catalytic activity was evaluated by the addition of different amounts of methanol (0, 10%, 20%, 40%, 60%, 80%, and 100% *v/v*) in the reactants. The absorbance of the supernatant at 485 nm was measured after 2 h.

### 2.6. Preparation of MnO_2_@CeO_2_/GCE

A total of 5 mg of MnO_2_@CeO_2_ nanozyme and 25 mg of PEI were added into 5 mL double distilled water and sonicated for 30 min. The GCEs were polished with 0.05 µm and 0.3 µm alumina pastes, then washed with distilled water and dried at 26 °C. A total of 5 µL of 1 mg/mL MnO_2_@CeO_2_ nanozyme suspension was dropped on the GCEs and then dried at room temperature. The dried modified electrode was used for the electrochemical detection of BPA and CC.

### 2.7. Electrochemical Experiments

A Tris buffer solution of 0.1 mol L^−1^, pH 7.0 was used as the electrolyte solution at room temperature. A three-electrode system was formed with the MnO_2_@CeO_2_/GCE electrode, with the GCE electrode as the working electrode, Pt wire as the main auxiliary electrode, and Ag/AgCl as the reference electrode. The size of the electrolytic cell was 5 mL, which was purchased from Guangzhou Saios Chemical Instrument Co., Ltd., Guangzhou, China. DPV was used with a pulse amplitude of 50 mV, a pulse width of 0.05 s, a potential increment of 4 mV, a pulse cycle time of 0.5 s, a sensitivity of 1e^−5^ A V^−1^, and a scanning in the negative direction at a potential in the range of 0.2–1.4 V with a scanning speed of 100 mV s^−1^, and the DPV curve was recorded.

### 2.8. High-Performance Liquid Chromatography (HPLC) Analyses

The HPLC analysis of BPA was conducted using a HPLC instrument (Thermo Fisher UltiMate 3000, Waltham, MA, USA) equipped with a reversed-phased column Syncronis C18 column (100 mm × 2.1 mm, 1.7 μm) maintained at a constant temperature of 30 °C with a diode array detector (DAD) set at 280 nm. The analysis involved the injection of 10 μL of 0.22 μm membrane-filtered samples at a flow rate of 0.2 mL/min, and the solvents consisted of a methanol–water mixture in a ratio of 65:35 (*v/v*).

A HPLC system (Prominence LC-20A, Shimadzu, Japan) with an analytical column Venusil MP C18 (4.6 mm × 250 mm, 5 μm) was used at a constant temperature of 35 °C. The isocratic elution was performed using a 5 mM ammonium acetate−1‰ formic acid solution (solvent A) and methanol (solvent B) in a ratio of 30:70 (*v/v*). The injection volume was 10 μL of 0.22 μm membrane-filtered samples, with a flow rate of 1 mL/min.

## 3. Results

### 3.1. Characterization of CeO_2_ NRs and MnO_2_@CeO_2_ NRs

**Figure 1:**
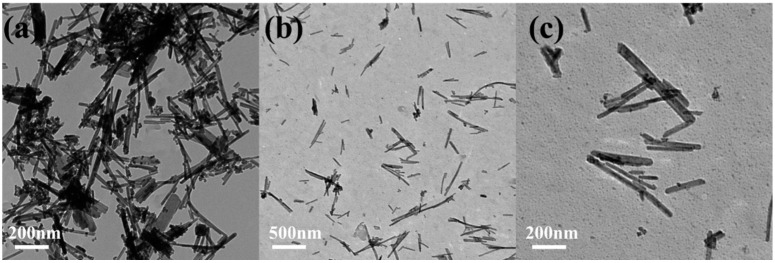
TEM images of (**a**) CeO_2_, (**b**) MnO_2_@CeO_2_ (500 nm), (**c**) MnO_2_@CeO_2_ (200 nm).

**Figure 2:**
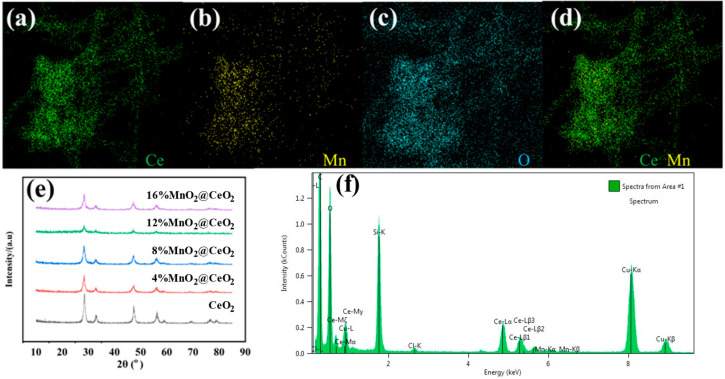
(**a**–**d**) Elemental mapping of MnO_2_@CeO_2_ NRs; (**e**) XRD pattern of MnO_2_@CeO_2_ NRs with different content of MnO_2_; (**f**) EDX analysis of MnO_2_@CeO_2_ NRs.

Figure 1 depicts TEM images of CeO_2_ NRs and MnO_2_@CeO_2_ NRs at different scales. It can be observed that CeO_2_ NRs exhibit a well-dispersed, rod-like structure with an average diameter of 8 nm. The elemental maps of Ce, Mn, and O in MnO_2_@CeO_2_ NRs are presented in Figure 2a–d. The maps show homogeneous distribution of O, Ce, and Mn throughout the nanorods, suggesting successful incorporation of Mn into the Ce-based nanorods. XRD was used to characterize the crystal phase of CeO_2_ NRs and MnO_2_@CeO_2_ NRs with varying mass ratios (Figure 2e). The diffraction peaks observed at 2θ angles of 28.5°, 33.1°, 47.5°, and 56.3° correspond to the (111), (200), (220), and (311) crystalline phases. As the amount of MnO_2_ increased, the diffraction peaks shifted towards the lower angle region, indicating that Mn entered the CeO_2_ lattice and resulted in structural defects of CeO_2_ NRs.

Figure 2f presents EDX images of the synthesized nanorods, showing the presence of Mn, O, and Ce, which confirms the successful synthesis of MnO_2_@CeO_2_ NRs.

### 3.2. Comparation of the Catalytic Ability of MnO_2_@CeO_2_ and Laccase

**Figure 3:**
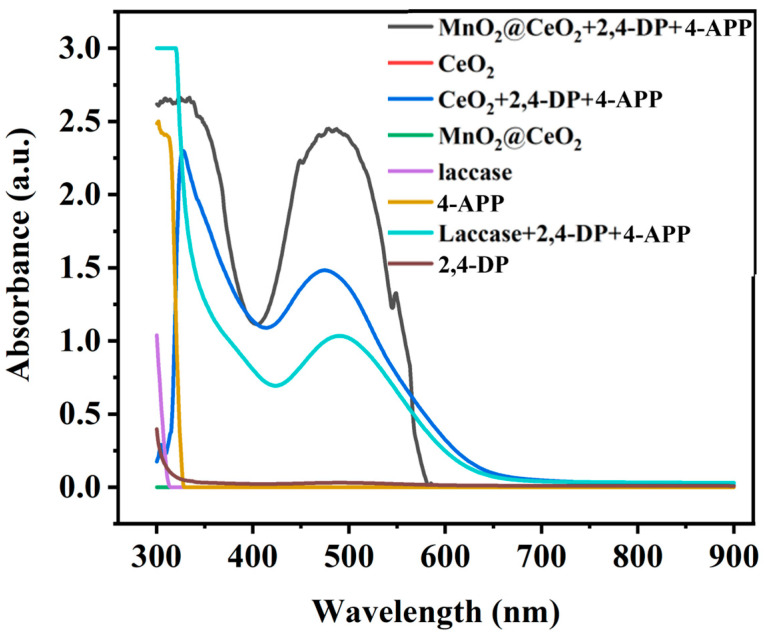
Absorbance spectrums of different catalytic systems.

The absorption spectra of various catalytic systems are presented in Figure 3. Laccase can catalyse the oxidation of 2,4-DP by simultaneously reducing molecular oxygen to water. As 2,4-DP is oxidized, the color of the solution gradually changes from colorless to red due to the reaction of the oxidation products of the phenolic pollutant with 4-APP to form a red adduct. As a result, the absorbance at 485 nm gradually increases (Figure 3, green curve). In the presence of CeO_2_ NRs, 2,4-DP, and 4-APP, a similar absorption peak at 485 nm was observed, indicating the laccase-like activity. In contrast, the absorption peaks in the MnO_2_@CeO_2_ system increased by 300% compared to CeO_2_ NRs, suggesting a significant enhancement of laccase-like activity. These findings imply that MnO_2_ can effectively improve the catalytic activity of CeO_2_ NRs. The other solutions containing the substrate without laccase or nanozyme did not show an absorption peak at 485 nm. These results confirm that the MnO_2_@CeO_2_ nanozyme possesses prominent laccase-mimicking catalytic performance.

**Figure 4:**
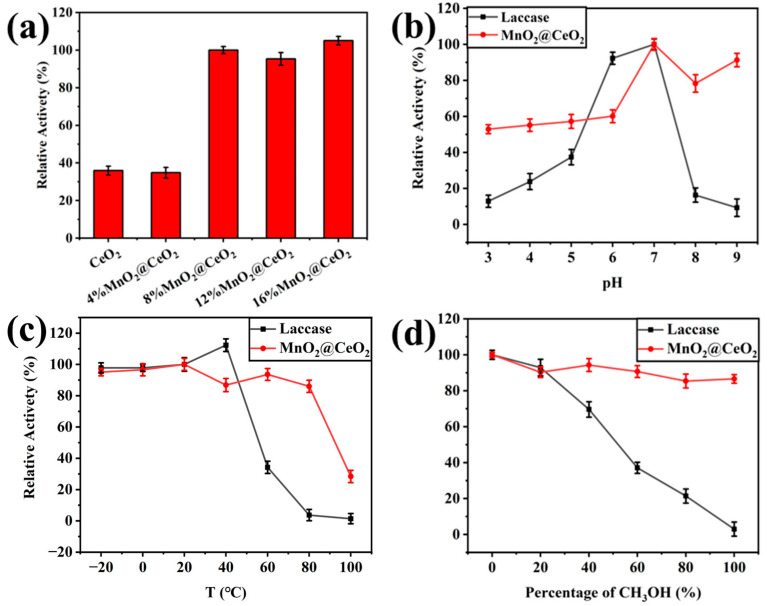
Relative activity of the MnO_2_@CeO_2_ nanozyme with increasing the content of Mn (**a**); rel-ative activity of the MnO_2_@CeO_2_ compared with the same mass concentration of laccase at different pH (**b**), temperature (**c**), and in different CH_3_OH solutions (**d**).

Figure 4a shows the relative activity of the MnO_2_@CeO_2_ nanozyme with different Mn contents at pH 7.0, 20 °C and without methanol. As the Mn content was increased from 4% to 8%, a dramatic enhancement in the activity of the MnO_2_@CeO_2_ nanozyme was observed, indicating that the incorporation of MnO_2_ effectively augmented the activity of the nanozymes. However, further increasing the Mn content from 8% to 16% did not yield any statistically significant differences in enzyme activity. The minor variations observed may be attributable to experimental error. Therefore, MnO_2_@CeO_2_ NRs with 8% Mn were used for the following experiments considering material consumption.

The catalytic activities of laccase and MnO_2_@CeO_2_ nanozyme were compared at different pH values (Figure 4b) and temperatures (Figure 4c) to verify the robust adaptability of the nanozyme. The effect of the pH value on catalytic activity was investigated at 20 °C without methanol. The catalytic activity of the laccase and MnO_2_@CeO_2_ nanozyme at pH 7.0 was considered as 100%, respectively. As depicted in Figure 4b, the MnO_2_@CeO_2_ nanozyme exhibited better catalytic activity in the range from pH 3.0 to 9.0 than laccase.

Testing at pH 7.0 without methanol, the catalytic activities of the laccase and MnO_2_@CeO_2_ nanozyme at 20 °C were considered as 100%, respectively. Laccase had the highest catalytic activity at 40 °C, and its activity sharply decreased as the temperature increased from 40 to 100 °C, almost becoming inactive at temperatures from 80 to 100 °C. In contrast, the relative activity of the MnO_2_@CeO_2_ nanozyme remained consistent from 20 to 80 °C and could still retain about 30% of the catalytic activity at 100 °C. These results indicate that the MnO_2_@CeO_2_ nanozyme exhibits better catalytic ability at high temperatures, as shown in Figure 4c.

To investigate the effect of organic solvent, different concentrations of methanol solution (0, 20%, 40%, 60%, 80%, and 100% *v/v*) were utilized under experimental conditions of pH 7.0 and 20 °C. As depicted in Figure 4d, the catalytic activity of laccase decreased as the methanol concentration increased. In 100% methanol solution, laccase was entirely inactivated. However, the MnO_2_@CeO_2_ nanozyme retained 90% of its activity in 100% methanol solution.

These results demonstrate that MnO_2_@CeO_2_ nanozymes have robust adaptability in different conditions.

### 3.3. Kinetic Parameters of NRs and Laccase

**Figure 5:**
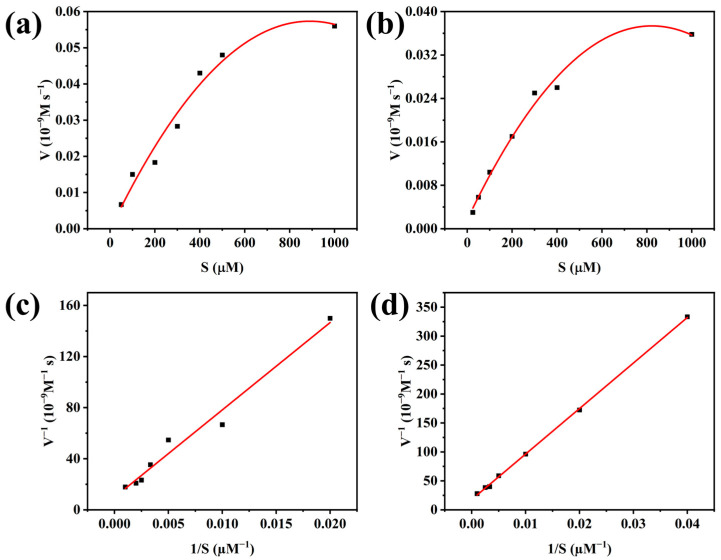
Steady-state kinetics of laccase (**a**,**c**) and MnO_2_@CeO_2_ nanozyme (**b**,**d**) by the catalytic oxidation with different concentrations of 2,4-DP. (**c**,**d**) are double-reciprocal plots of (**a**,**b**).

The kinetic parameters of the MnO_2_@CeO_2_ nanozyme and laccase were investigated by studying the oxidation of different concentrations of 2,4-DP (Figure 5). The *K*_m_ value for the MnO_2_@CeO_2_ nanozyme was 0.7 mM, while that of laccase was 0.4 mM (shown in Table 1). The former showed nearly a 2-fold higher *K*_m_ than laccase, indicating weaker substrate-binding ability compared to the natural enzyme. Natural enzymes have flexible active sites, whereas the nanozyme is conformationally rigid. Hence, natural laccase exhibited stronger affinity towards the substrate than the MnO_2_@CeO_2_ nanozyme. The *V*_max_ value of the MnO_2_@CeO_2_ nanozyme was 6 µM, while that of laccase was 3 µM, indicating a faster reaction rate for the MnO_2_@CeO_2_ nanozyme.

### 3.4. Detection of Phenols Based on MnO_2_@CeO_2_/GCE

**Figure 6:**
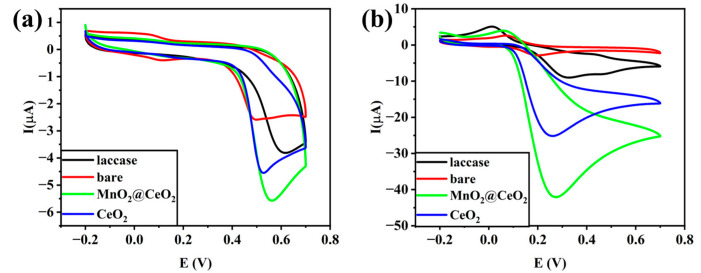
Cyclic voltammograms of bare GCE, laccase/GCE, MnO_2_@CeO_2_/GCE, and CeO_2_/GCE in 0.1 M pH 7.0 Tris containing BPA (**a**) and CC (**b**) with scan rate of 50 mV s^−1^.

Cyclic voltammograms of bare GCE, laccase/GCE, MnO_2_@CeO_2_/GCE, and CeO_2_/GCE in 0.1 M pH 7.0 Tris containing BPA and CC at a concentration of 10^−5^ M are shown in Figure 6. The peak currents of MnO_2_@CeO_2_/GCE for BPA and CC were much higher than those of the other electrodes, indicating excellent electrocatalytic activity of the MnO_2_@CeO_2_ nanozyme. The oxidation peaks observed in cyclic voltammograms are typically attributed to the oxidation of phenolic compounds. Specifically, laccase or nanozymes facilitate the oxidation of the hydroxyl group (-OH) in phenol, transferring electrons to molecular oxygen to generate the oxidized form of phenol (quinone) along with water. This reaction produces a signal at the electrode, which is manifested as an oxidation peak in the cyclic voltammogram.

**Figure 7:**
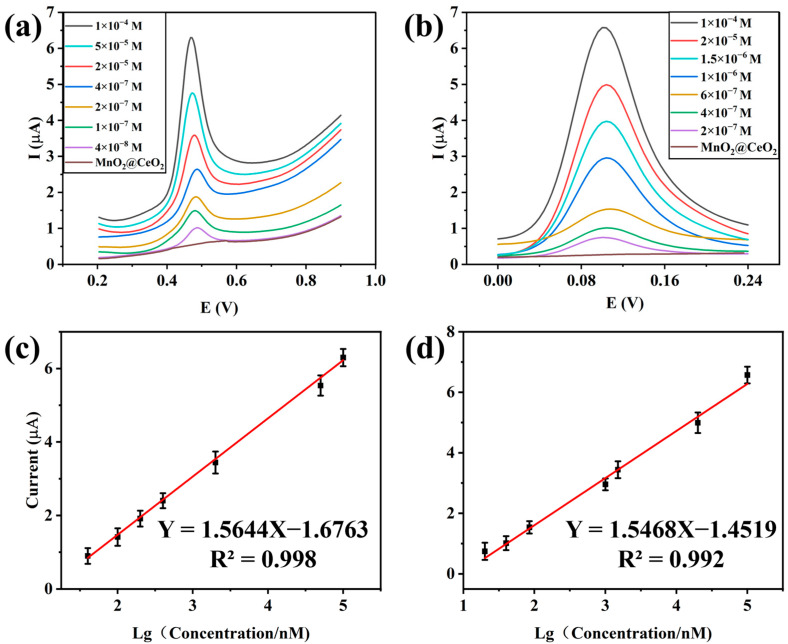
DPV responses of MnO_2_@CeO_2_/GCE with different concentrations of BPA (**a**) and CC (**b**); the linear calibration plot of BPA (**c**) and CC (**d**).

DPV was used for the effective detection of BPA and CC in 0.1 M Tris buffer solution with a scan rate of 0.05 v/s. Under the optimized conditions of MnO_2_@CeO_2_ NRs shown in Figure 4, the oxidation peak current increased linearly with increasing BPA and CC concentrations (Figure 7a,b). There were linear relationships between the peak currents and the logarithm of the concentrations for BPA in the range of 4 × 10^−8^ to 1 × 10^−4^ M and for CC in the range of 2 × 10^−7^ to 1 × 10^−4^ M. The current change was also linearly correlated with the logarithm of BPA and CC concentrations within these ranges. The regression equations were I = 1.56 log BPA − 1.68 (R^2^ = 0.998), I = 1.55 log CC − 1.45 (R^2^ = 0.992). The detection limits (S/N = 3) for BPA and CC were 1.2 × 10^−8^ M and 7.3 × 10^−8^ M, respectively. The response time for completing a single DPV reaction is 10 s. The well-defined anodic peaks of BPA and CC indicate the excellent electrocatalytic performance of MnO_2_@CeO_2_ nanozymes.

### 3.5. Selectivity, Reproducibility, and Stability

**Figure 8:**
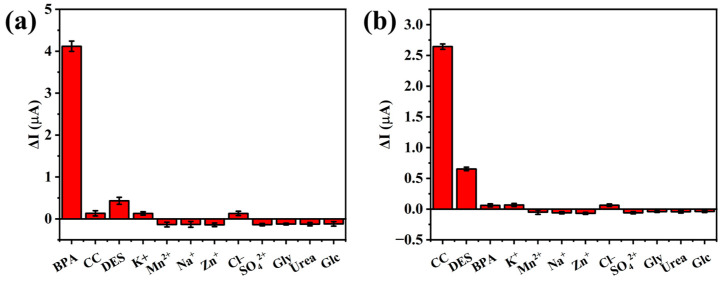
The selectivity of the proposed system towards BPA (**a**) and CC (**b**) detection.

To investigate the selectivity of this method, some common metal ions and potential substances, including cations (K^+^, Mn^2+^, Na^+^, Zn^2+^), anions (Cl^−^, SO_4_^2−^), glycine (Gly), urea, and glucose (Glc) were tested at concentrations 10 times higher than that of BPA and CC, which were both at 10^−5^ M. As shown in Figure 8, no significant changes were observed after the addition of interferents, indicating the high selectivity of the method.

**Figure 9:**
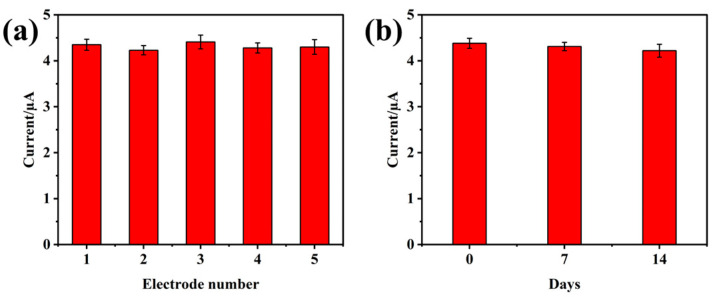
(**a**) Reproducibility and (**b**) stability of the electrodes.

In addition, we also evaluated the stability and reusability of the electrodes. First, five identical electrodes were used to test 0.1 mM BPA, and the relative standard deviation was 1.59%, indicating excellent reproducibility (Figure 9a). Subsequently, the electrode was stored at room temperature for a certain period and then used to detect 0.1 mM BPA. As shown in Figure 9b, after 14 days, the peak current decreased by only 1.86% compared to its initial value. These results demonstrate that the electrodes exhibit good stability.

### 3.6. Detection of BPA and CC in Red Wine

To evaluate the practicality of this method, red wine purchased from a supermarket was selected as a sample. Different concentrations of BPA and CC (5, 50, and 500 μmol/L, respectively) were added to the red wine, and the recoveries and relative standard deviation (RSD) were calculated (shown in Table 2). The results indicated that this method has excellent accuracy and precision and can be used for the detection of phenolic compounds in practical samples.

## 4. Conclusions

In summary, the MnO_2_@CeO_2_ nanozyme with laccase-like activity was successfully prepared and exhibits excellent stability and environmental suitability under various harsh conditions compared with laccase. Furthermore, the MnO_2_@CeO_2_ nanozyme was used to fabricate a modified electrode for electrocatalytic detection, which showed good accuracy with a low detection limit and high selectivity. This work provides a novel strategy for developing electrochemical methods for the detection of phenolic compounds in practical samples based on the MnO_2_@CeO_2_ nanozyme.

## Figures and Tables

**Table 1:** Kinetic parameters of MnO_2_@CeO_2_ nanozyme and laccase for 2,4-DP at 37 °C.

Catalyst	*K_m_* (mM)	*V_max_* (mM min^−1^)
MnO_2_@CeO_2_	0.7	6
Laccase	0.4	3

**Table 2:** Recoveries and RSD of BPA and CC in actual samples.

Analytes	Added (μM)	This Work			HPLC		
		Found (μM)	Recovery (%)	RSD (%)	Found (μM)	Recovery (%)	RSD (%)
BPA	5	4.9	98.0	2.0	4.6	93.0	4.7
	50	48.8	97.6	2.4	46.2	92.4	5.0
	500	491.3	98.2	3.1	542.5	108.5	4.2
CC	5	5.1	102.0	2.8	5.3	106.0	5.9
	50	49.1	98.2	2.3	45.9	91.8	6.3
	500	488.5	97.7	3.0	450.5	90.1	5.6

## Data Availability

The original contributions presented in the study are included in the article; further inquiries can be directed to the corresponding author.
